# Supplementary material for: CBP and p300 Jointly Maintain Neural Progenitor Viability but Play Unique Roles in the Differentiation of Neural Lineages
Source: Cells. 2022 Dec 18;11(24):4118. doi: 10.3390/cells11244118 (PMC9777331; doi:10.3390/cells11244118)
Supplement: Supplementary file 1 [file cells-11-04118-s001.zip › Figure S1-S2.pdf]

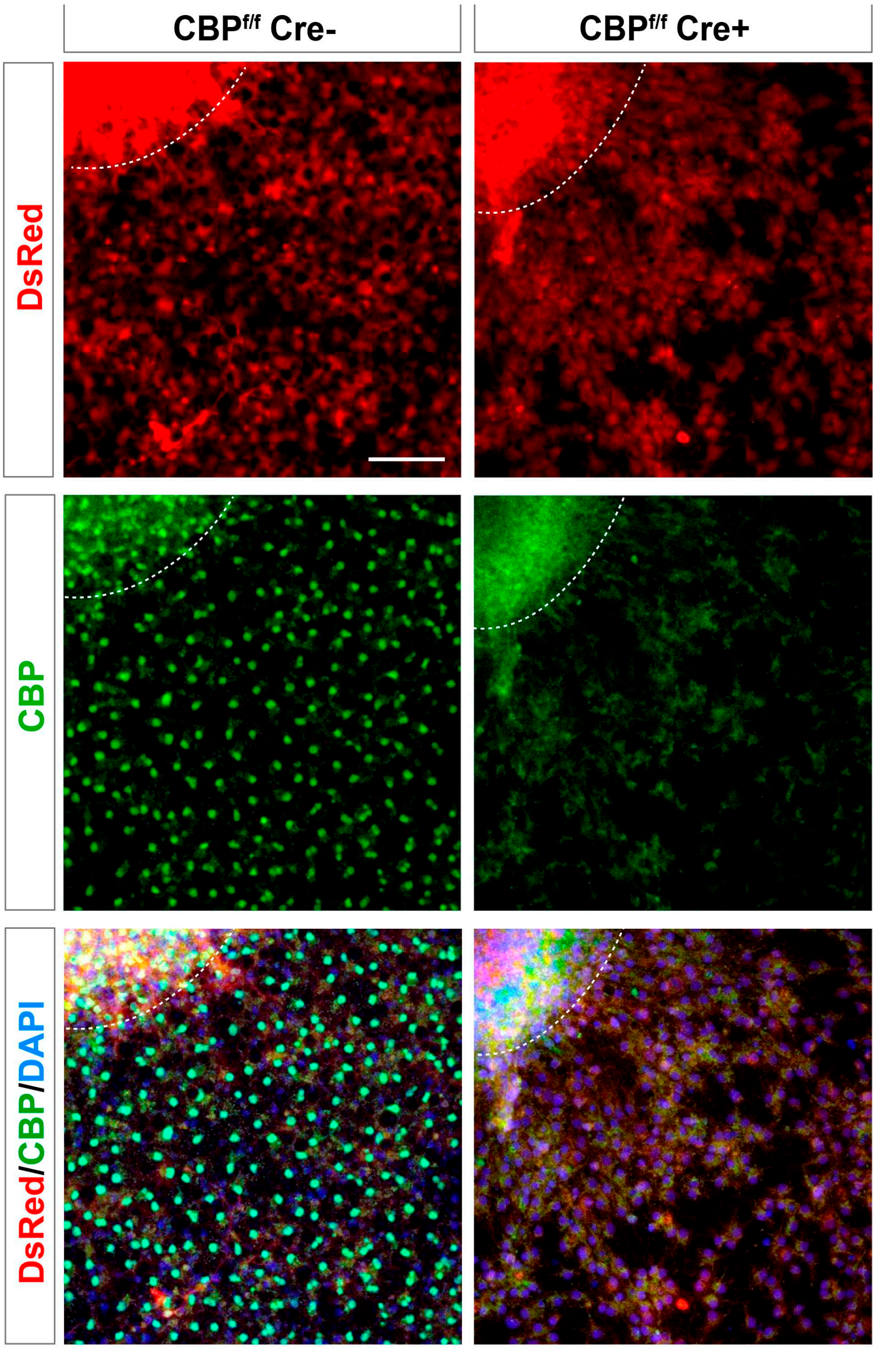

Figure S1. Differentiating CBP$^{f/f}$ neurospheres infected with Cre-recombinase encoding viruses do not express CBP. Representative images of differentiating secondary neurospheres infected with control (DsRed) or Cre-Recombinase-encoding Lentiviruses stained with antibodies specific for CBP and contrastained with DAPI. Scale bars: 100 µm

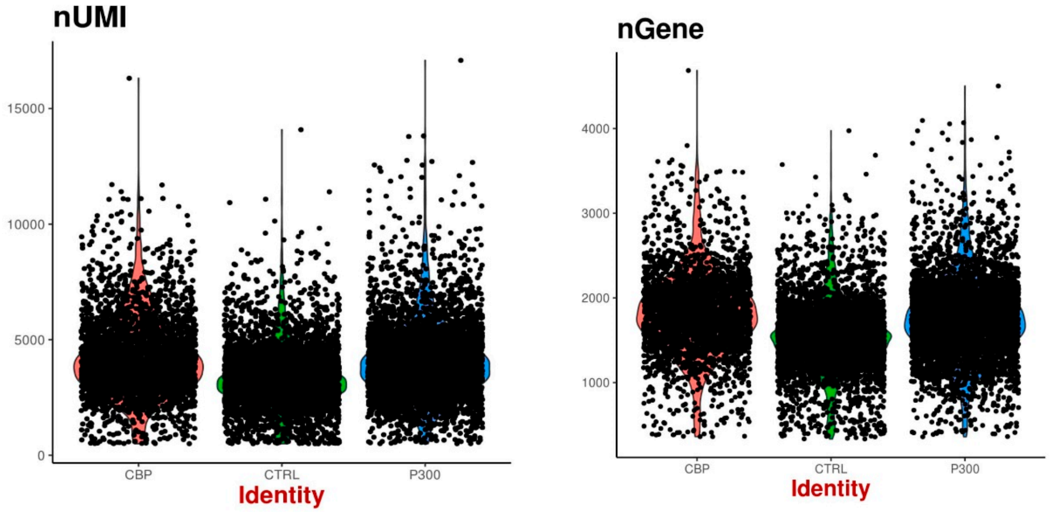

Figure S2. Sensitivity analysis of snRNA-seq. Violin plots show the distribution of the number of transcripts (left, scored by UMIs) and genes (right) detected per cell for differentiating neurospheres from control (CTRL), CBP$^{f/f}$ (CBP) and p300$^{f/f}$ (P300) neurospheres. UMIs: unique molecular identifiers.
